# Treatment of lactational mastitis with Gualou Xiaoyong soup and painless lactation promoting technique: A case series

**DOI:** 10.1097/MD.0000000000036384

**Published:** 2023-12-01

**Authors:** Lihua Jin, Huijun Ye, Yi Yang, Jiali Liu, Ruilan Li, Huiling Zheng

**Affiliations:** a Department of Gynaecology and Obstetrics, The Second Affiliated Hospital of Zhejiang Chinese Medicine University, Hangzhou City, China.

**Keywords:** case report, case series, Gualou Xiaoyong soup, lactation mastitis, lactation promoting technique, traditional Chinese medicine treatment techniques, treatment

## Abstract

**Background:**

Lactational mastitis is a common and frequently disease in clinical practice, characterized by acute inflammation of the mammary ducts and surrounding connective tissues. The main manifestations are damage to the mammary gland acini, edema, and invasion of inflammatory cells. If not treated properly, it may lead to the formation of breast abscesses, or even sepsis, septic shock, and chronic inflammation of the breast, which may cause the disease to persist or recur multiple times, so that the patients suffer extreme pain, and the health of both the mother and child are directly affected. This disease not only causes suffering for women but also may result in the cessation of breastfeeding. Therefore, rapid and effective treatment is particularly important.

**Case summary:**

We report 3 cases of lactation mastitis patients showing good clinical efficacy after being treated with the Chinese medicine Gualou Xiaoyong soup and painless lactation promoting techniques. Gualou Xiaoyong soup combined with painless lactation promotion techniques can significantly reduce and eliminate the clinical symptoms of patients in the short term, and rapidly restore inflammatory indicators such as total white blood cells, neutrophils, C-reactive protein, and procalcitonin to normal levels. The patchy low echo area of the breast under B-ultrasound also disappears quickly. Therefore, we believe that this method is a good way to treat lactational mastitis and is worthy of clinical reference and research. However, this study has certain limitations: this study lacks a large sample of prospective controlled studies. Next, we will continue to collect relevant cases and conduct prospective case randomized controlled clinical studies.

**Conclusion:**

The treatment of lactation mastitis with Gualou Xiaoyong soup and painless lactation promoting techniques can achieve good clinical results.

## 1. Introduction

Lactational mastitis is a common and frequently occurring disease in clinical practice,^[1]^ characterized by acute inflammation of the mammary ducts and surrounding connective tissues. The main manifestations are damage to the mammary gland acini, edema, and invasion of inflammatory cells.^[2]^ It is estimated that about 33% of lactating women have a history of this disease.^[3]^ The disease is associated with symptoms such as breast pain, swelling, and rubefaction; fever; fatigue; and flu-like symptoms, with axillary lymph nodes enlarging and inflammatory cell counts are increasing. Inadequate treatment of lactation mastitis can lead to the formation of breast abscess, sepsis, and chronic breast inflammation, and can also directly affect the health of both the mother and child.^[2]^ The etiology of this disease is mostly related to the microbiota in the nasopharynx of newborns and infants, such as *Staphylococcus aureus* and *Streptococcus*, which infect the breast through damaged epithelial cells of the nipple-areolar complex.^[4–8]^ This disease not only causes suffering for women but also may result in the cessation of breastfeeding.^[4]^ Current treatment methods are mainly promoting lactation to relieve milk accumulation, hot-compression, and controlling inflammation.^[6]^

Modern medicine mostly uses intravenous infusion of antibiotics for anti-infective treatment, which cannot solve the problem of milk accumulation, leading to poor efficacy and long course of treatment, requiring cessation of breastfeeding, being prone to antibiotic-resistant strains, and causing reduced milk production by lactating mothers and the formation of chronic breast lumps.^[7]^ In contrast, traditional Chinese medicine treatment techniques combines Gualou Xiaoyong soup with painless lactation promotion, has advantages such as shorter courses of treatment, no need to stop breastfeeding, and rapid symptom improvement. In this study, Gualou Xiaoyong soup combined with painless lactation promoting techniques was used to treat lactation mastitis. It was found that it had good clinical efficacy, not only could quickly improve the clinical manifestations of breast mass, local skin redness, swelling, heat, pain, fever, etc, but also had a good improvement effect on various laboratory and imaging indicators.

This study was approved by the Medical Ethics Committee of the Second Affiliated Hospital of Zhejiang University of Traditional Chinese Medicine, and the patients gave informed consent.

## 2. Results/case presentation

### 2.1. Case 1

Female Chinese, 32, “Right breast swelling and pain with local skin redness and swelling for 5 days, left breast swelling and pain for 1 day,” first visited on May 17, 2022. The patient underwent a successful cesarean section in another hospital due to “fetal distress”56 days ago. Five days ago, she developed right breast tenderness without fever, with reddish local skin. One day ago, left breast tenderness became more prominent. Currently, the patient feels significant discomfort and pain bilaterally, with the pain rating (visual analogue score rating) was 5. The patient has no other medical history and no known allergies. Body temperature of 36.5°C, pulse rate 80 beats/min, and respiratory rate 20 breaths/min. A 10 cm * 9 cm hard mass was palpable in the right breast, with tenderness and slightly red skin. A 3 cm * 3 cm mass was palpable in the left breast, with tenderness and no redness or swelling. Breast ultrasound showed lactating breast with cysts in both breasts and multiple hypoechoic areas (1.1 cm * 0.5 cm on the left and 0.9 cm * 0.6 cm on the right), the total number of white blood cells (WBC), the percentage of neutrophils (%) and C-reactive protein (CRP) in blood routine tests were normal before and after treatment, but procalcitonin decreased from 1.14 to 0.43 mg/L after treatment. Before treatment, *Staphylococcus epidermidis* was cultured in the milk of case 1 and it resistant to penicillin and Cephalosporin. After 1 week of treatment, the bacterial culture in milk was negative.

### 2.2. Case 2

Female Chinese, 36, “6 days after normal delivery, 3 days of breast tenderness,” first visited on May 4, 2022. A postpartum woman who gave birth vaginally 6 days ago without complications. Three days ago, she began to experience significant breast pain that was difficult to bear, accompanied by chills, fatigue, poor appetite, and poor mental state. She had no fever, the visual analogue score pain score was 9. The patient had a history of IgA nephropathy for over 10 years. Body temperature of 38.5°C, a palpable 10 cm * 10 cm sized hard lump on the outer right breast with a 10 cm * 10 cm-sized locally red and purple area, high skin temperature, felt obvious tenderness in the area. Breast ultrasound showed bilateral axillary lymph nodes in lactating period.

### 2.3. Case 3

Female Chinese, 30, “13 months after normal delivery, breast tenderness and fever for 1 day,” first visited on June 9, 2022. A woman who delivered vaginally over 13 months ago developed breast pain after self-weaning at home yesterday. She had a fever yesterday, and body temperature was 38.9°C early this morning. Body temperature decreased to 37.8°C after she drank more water at home, but she still felt weak and had sore limbs. There was no loss of appetite, nausea or vomiting. The patient has no other medical history and no known allergies. Body temperature of 37.8°C, a palpable 7 cm * 5 cm sized hard lump with obvious tenderness and redness on the lower inner quadrant of the right breast and localized fever. Purulent discharge can be seen with lactation promoting for the right breast, milk discharge from the left breast was normal. Breast ultrasound showed bilateral low echo area in lactating period, with sizes of 1.1 cm * 0.7 cm and 2.0 cm * 1.1 cm, suggesting bilateral breast inflammatory changes.

The clinical diagnosis of all 3 cases was lactational mastitis. All patients were treated with the following methods:

1. Oral administration of Chinese medicine:
2. Gualou Xiaoyong soup: Chaihu 9g, Gualoupi 15g, Sigua Luo 15g, Banbianlian 15g, Pugongying 15g, Chishao 15g, Tongcao 6g, Taoren 10g, Chao Yiyiren 30g, Chixiaodou 20g, Danggui 15g, Chao Baizhu 15g, Niubangzi 15g, Jinyinhua 20g, Lianqiao 15g, Fuling 15g, Lulutong 10g, Baizhi 15g, and Zaojiaoci 15g. The prescription is decocted in 400ml of water, and the decoction is taken warm in the morning and afternoon, with 200ml each time, 1 prescription a day.
3. Painless lactation promotion:
4. Press each acupoint of Shanzhong, Ruzhong, Rugen, Qimen, Shaohai, Chize, Tianchi, Tianxi for 1 to 2 minutes. Then perform breast massage to eliminate the accumulated milk: place the thumb and index finger of one hand on the black-white border of the areola, use a downward and inward pressing motion to express out the front milk, then apply massage oil to the hand, use the fingertips or the edge of the hand to gently rub the lumps in the breast 3 times, gently push the milk out once in the direction of the nipple. Perform this massage for 20 to 30 minutes for each breast.

**Figure 1.**
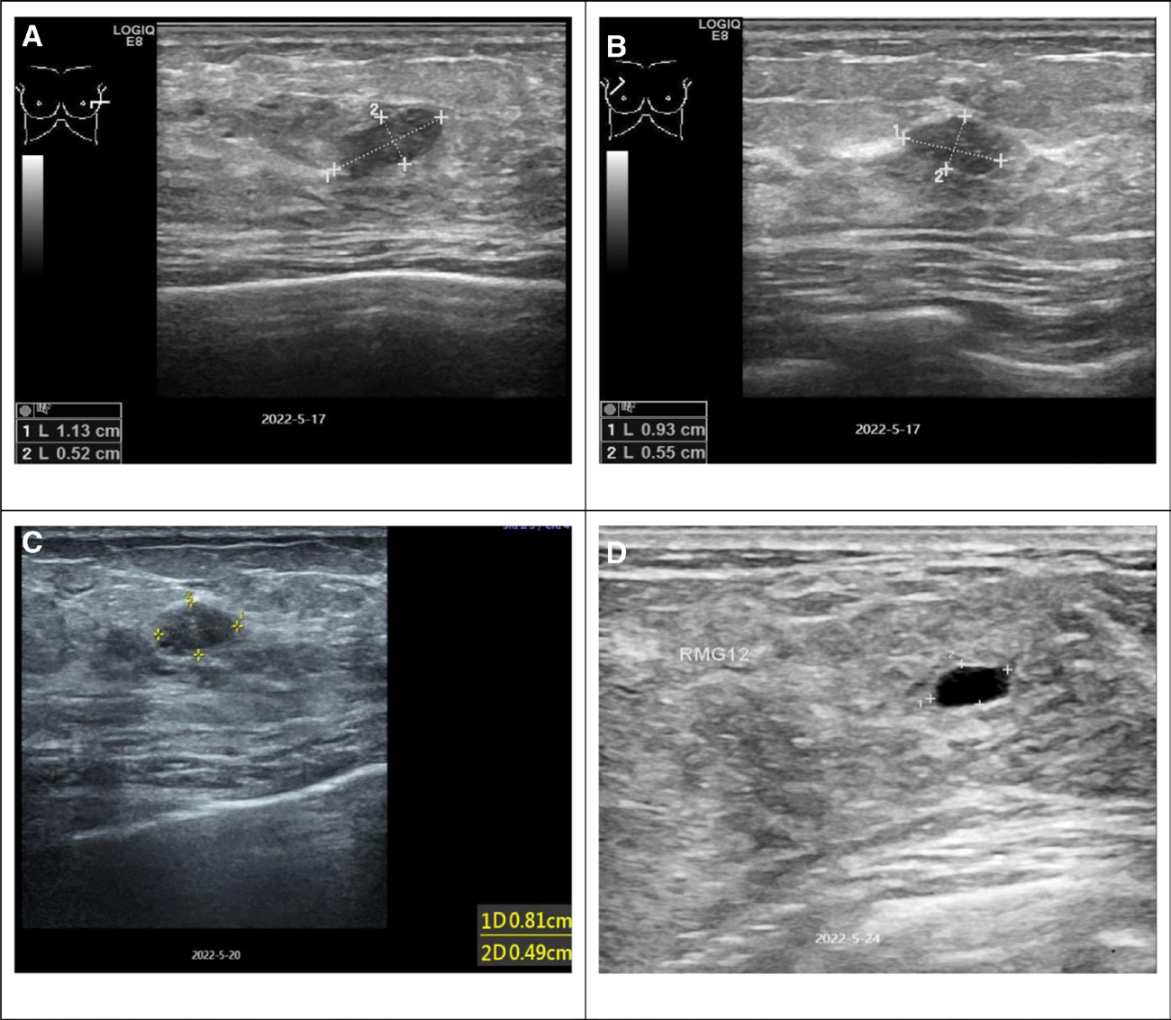
(A) On May 17, 2022: A low-echo area of 1.1 cm * 0.5 cm in the left breast. (B) On May 17, 2022: A low-echo area of 0.9 cm * 0.6 cm in the right breast. (C) On May 20, 2022: A low-echo area of 0.8 cm * 0.5 cm in the left breast. (D) On May 24, 2022: Normal lactating breast and breast cysts.

**Figure 2.**
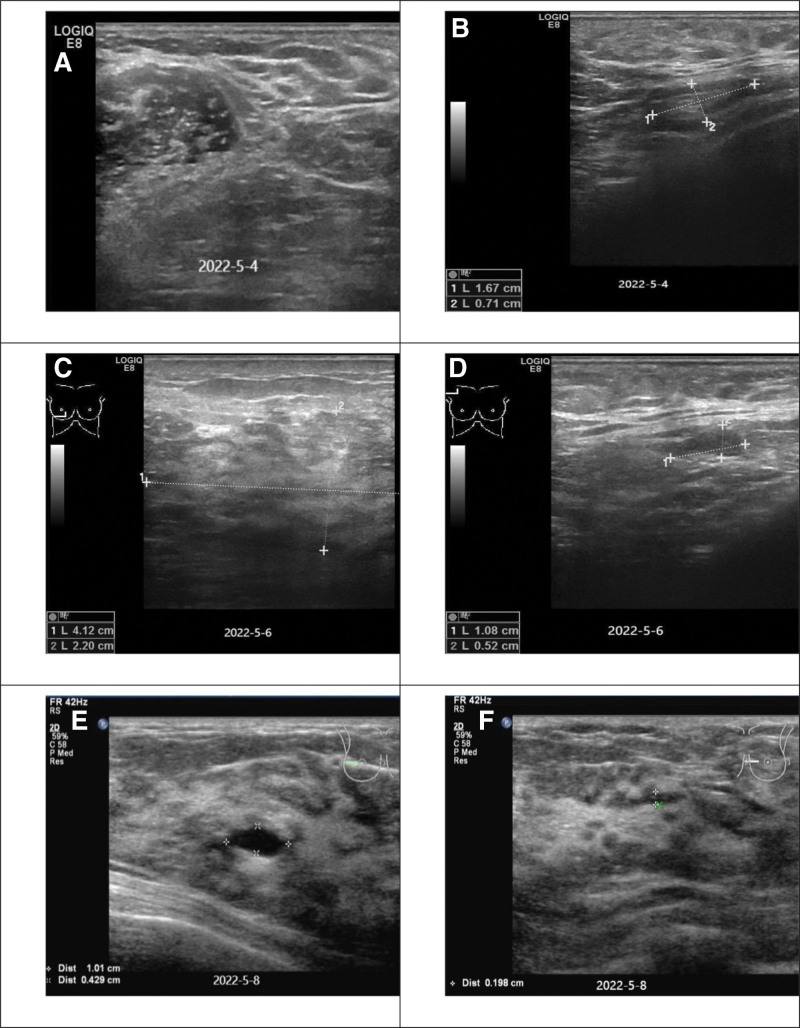
(A) On May 4, 2020: Multiple low-level echoes and echo-free areas of different sizes visible in both breasts. (B) On May 4, 2020: Underarm lymph nodes, with the larger one measuring approximately 1.7 cm *0.7 cm on the right. (C) On May 6, 2020: A patchy low-level echo area visible in the 8 to 10 o’clock direction of the right breast, with a range of 4.2 cm * 2.2 cm. (D) On May 6, 2020: A low-level echo nodule with a clear boundary visible on the right side, measuring approximately 1.1 cm * 0.5 cm. (E) On May 8, 2020: A breast cyst on the right side. (F) 2022.05.08: Normal lactating breast.

**Figure 3.**
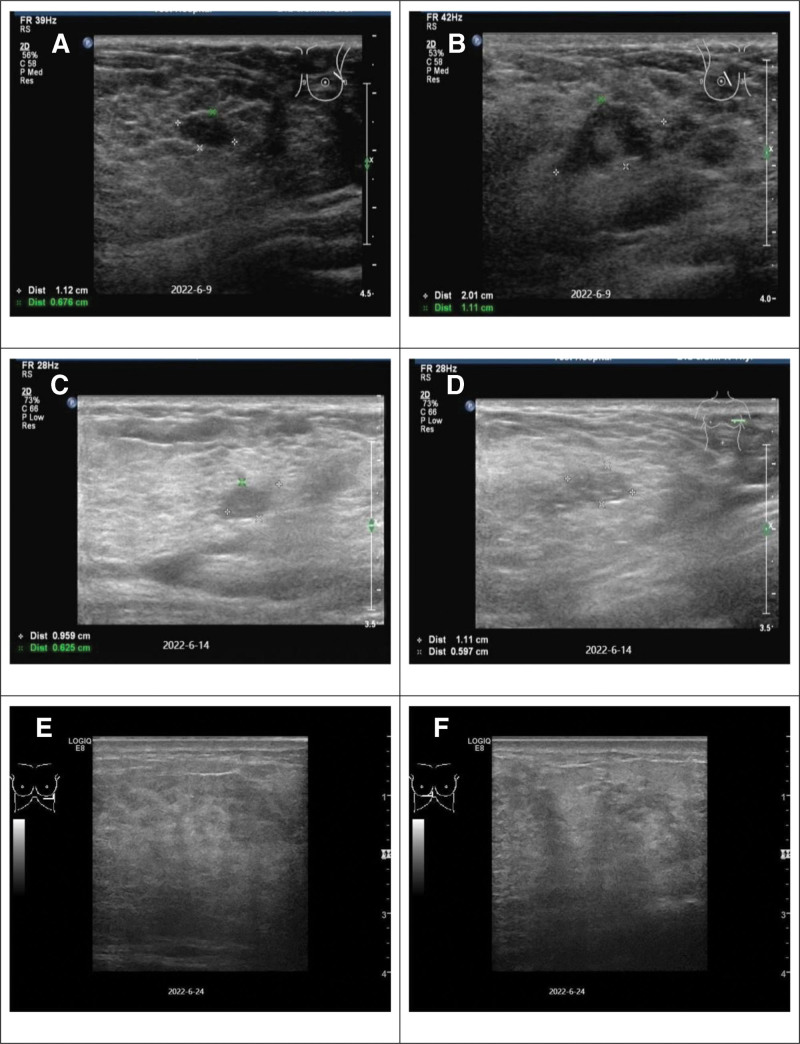
(A) On June 9, 2022: A low-echo area of approximately 1.0 cm * 0.7 cm detected in the 3 o’clock direction of the left breast. (B) On June 9, 2022: A low-echo area of approximately 2.0 cm * 1.1 cm detected in the upper inner quadrant of the right breast. (C) On June 14, 2022: A low-echo area of approximately 1.0 cm * 0.6 cm detected in the 3 o’clock direction of the left breast. (D) On June 14, 2022: A low-echo area of approximately 1.0 cm * 0.6 cm detected in the upper inner quadrant of the right breast. (E and F) On June 24, 2022: The mammary glands of both breasts were normal, consistent with lactational changes.

The patients of all 3 cases had recovered well and was followed up after 1 month with no recurrence reported, the detailed results are shown in Tables 1 and 2 and Figures 1–3.

**Table 1:** Laboratory indicators changes before and after treatment in case 1 to 3.

	Date	WBC (*10ˆ9/L)	NE (%)	CRP (mg/L)	PCT (mg/L)
Case 1	2022.05.17	7	53.6	1.14	0.02
	2022.05.20	5.8	51.0	0.52	0.02
	2022.05.24	6.7	49.6	0.43	<0.02
Case 2	2022.05.04	18.1	92.7	36.17	0.06
	2022.05.06	11.1	88.8	164.85	0.06
	2022.05.08	8.9	77.8	43.75	0.02
Case 3	2022.06.09	21.2	84.5	24.33	0.05
	2022.06.14	8.0	61.1	7.31	0.04
	2022.06.24	10.5	70.2	0.64	0.03

CRP = C-reactive protein, NE = neutrophils, PCT = procalcitonin, WBC = white blood cells.

**Table 2:** Bacterial culture results and drug resistance before and after treatment in case 1 to 3.

	Date	Milk bacterial culture results	Penicillin resistance (Y/N)	Cephalosporin resistance(Y/N)
Case 1	2022.5.17	*Staphylococcus epidermidis*	Y	Y
	2022.5.20	*S epidermidis*	Y	Y
	2022.5.24	*No bacterial growth and fungi were found after 2 d of culture*	/	/
Case 2	2022.5.4	*Streptococcus viridans*	N	N
	2022.5.6	*S viridans, Staphylococcus aureus*	N	N
	2022.5.8	*No bacterial growth and fungi were found after 2 d of culture*	/	/
Case 3	2022.6.09	*Staphylococcus hominis*	Y	Y
	2022.6.14	*S hominis*	Y	Y
	2022.6.24	*No bacterial growth and fungi were found after 2 d of culture*	/	/

## 3. Discussion

It is well known that lactational mastitis may directly lead to cessation of breastfeeding. It is estimated that about 33% of lactating women have a history of this disease.^[3]^ This disease is a cellulitis of the interlobular connective tissue of the mammary gland, which mostly occurs in the second to third week after childbirth, while most reports show that 74% to 95% of cases occur within 12 weeks after childbirth.^[9,10]^ However, it may also occur at any stage of lactation, including the second year.^[9,10]^ The incidence of the disease is related to fatigue, excessive stress, blocked mammary ducts, inadequate feeding times, abnormal infant oral cavity (such as cleft lip or cleft palate), local milk accumulation, maternal malnutrition, use of manual breast pumps, community-acquired infections, breast injuries, and poor diet.^[11]^ The pathogenic bacteria typically involve *S aureus, Streptococcus*, and/or *Bacillus*.^[12]^ The severity of the condition varies, from mild symptoms to localized inflammation, redness, fever, and tenderness, and to more severe symptoms, including fever, abscess, and sepsis that require hospitalization. If the treatment is delayed or incomplete, breast abscess, mammary fistula, and recurrent mastitis may occur,^[10]^ which can be fatal.

Mastitis is an inflammatory disease of the mammary gland, which may or may not be accompanied by infection. If one of the following conditions is met, the diagnosis of acute lactational mastitis is made: localized redness of the breast, with or without elevated temperature; systemic inflammatory response, such as chills, headache, and fatigue; body temperature higher than 37.3°C or elevated WBC or neutrophil counts or CRP levels; patients with positive milk cultures.^[13]^ The pathogenic bacteria of mastitis involve *S aureus, Streptococcus,* and/or *Bacillus*, with the most common being *S aureus* and *S epidermidis*.^[12]^ Clinical doctors commonly use cephalosporins and penicillin antibiotics to treat lactational mastitis, as these 2 types of drugs can be used during lactation.^[14]^ However, the clinical efficacy seems to be not good, because many bacteria are resistant to these 2 types of antibiotics, just like the bacteria cultured from the milk of the 2 cases in this article (case 1 and case 3), both are resistant to cephalosporins and penicillin antibiotics. Other potentially sensitive antibiotics, such as quinolones, glycopeptides, and oxazolidinones, require postponing lactation when they are taken orally or intravenously. Therefore, antibiotics may not have an ideal clinical effect and may also affect breastfeeding. Using Chinese medicine Gualou Xiaoyong soup and painless lactation promotion technique to treat lactational mastitis has good clinical efficacy and does not require stopping breastfeeding.^[14]^

The Gualou Xiaoyong soup is an experienced description of our department, it has a good clinical effect on the treatment of lactational mastitis and breast abscess.^[14]^ The formula consists of the following herbs: Chaihu, Gualoupi, Niubangzi, Sigualuo, Banbianlian, Pugongying, Chishao, Tongcao, Taoren, Yiyiren, Chixiaodou, Danggui, Chaobaizhu, Jinyinhua, and Lianqiao. Chaihu and Gualoupi are used to soothe the liver and regulate qi; Chaobaizhu is used to strengthen the spleen and stomach; Niubangzi, Banbianlian, Pugongying, Jinyinhua, and Lianqiao are used to clear heat and detoxify, disperse nodules, and reduce swelling; Danggui, Chishao, and Taoren are used to cool and nourish the blood, promote blood circulation, and resolve stasis; Yiyiren and Chixiaodou are used to detoxify and promote suppuration; Sigualuo and Tongcao are used to unblock channels, disperse nodules, and promote lactation. All these herbs work together to soothe the liver and stomach, resolve phlegm, soften hardness, clear heat and detoxify, promote suppuration, and promote lactation. Researchers have studied these Chinese herbs and found that they contain active ingredients such as quercetin, luteolin, hesperetin, naringenin, and urushiol.^[15–17]^ Quercetin is a dietary flavonoid that has effects on inflammation, angiogenesis, and vascular inflammation, it has anti-inflammatory, antioxidant, and antifungal properties, and can enhance the transcriptional activity of NRF2-ARE by inhibiting the NF-κB signaling pathway, therefore regulating the production of the inflammatory precursor NO.^[15–17]^ Luteolin has a systemic anti-inflammatory effect and can regulate the TLR2 and TLR4 signaling pathways induced by *S aureus*. Hesperetin can inhibit the activation of the NF-κB inflammatory pathway and control the occurrence of inflammation.^[15–17]^ Naringenin can reduce the expression of IL-6, TNF-α, and ANGPTL2 in cells and prevent the occurrence of mouse mammary gland inflammation.^[15–17]^ Urushiol has a dose-dependent antibacterial effect on *S aureus, Escherichia coli*, and *Streptococcus agalactiae*.^[15–17]^

The primary treatment for mastitis is breast drainage to empty accumulated milk.^[18]^ Milk accumulation not only causes breast swelling, discomfort, and reduced milk production, but also serves as a good breeding ground for bacteria, exacerbating and recurring mastitis.^[18]^ Therefore, it is essential to empty the breast. Chinese medicine massage has the effect of unblocking the mammary ducts, promoting breast drainage, improving local blood and lymph circulation in the breast, promoting the absorption of edema and inflammatory products, and reducing internal pressure in the breast.^[19]^ Our painless lactation promotion techniques mainly combine massage of distant acupoints along the meridians and local breast massage, with excellent lactation promotion effects, and no pain or side effects for the patient. The operator first massages each acupoint among Shanzhong, Ruzhong, Rugen, Qimen, Shaohai, Chize, Tianchi, and Tianxi for 1 to 2 minutes, and then massages localized breast area. The breast meridians are closely related to the Ren channel, the Yangming Stomach Meridian, the Jueyin Pericardium Meridian, the Shaoyin Heart Meridian, the Taiyin Spleen Meridian, and the Jueyin Liver Meridian. Shanzhong is located on the Ren channel, which is the sea of Yin meridians and plays a vital role in regulating qi, widening the chest, and promoting blood and milk circulation. It is the key acupoint for lactation promotion. Rugen and Ruzhong belong to the Yangming Stomach Meridian, which is a meridian of abundant qi and blood. Massaging these 2 acupoints has the effect of regulating Yangming qi and blood, widening the chest, and promoting lactation. The liver meridian passes through the chest and encircles the nipple, so the nipple belongs to the liver, and Qimen is the gathering point of the liver meridian. The Taiyin, Jueyin, and Yinwei meridians converge at Qimen, which has the effect of tonifying the spleen, regulating the liver, and promoting qi circulation and blood flow. Chize is the junction point of the Hand Taiyin Lung Meridian, which has the effect of clearing heat and regulating the stomach, promoting meridian circulation, and relieving pain. Tianchi is located on the Hand Jueyin Pericardium meridian and is the junction point of the Hand and Foot Jueyin and Shaoyang meridians. It has the effect of promoting blood circulation, resolving stasis, regulating qi, and promoting meridian circulation. Tianxi is located on the Foot Taiyin Spleen meridian and has the effect of widening the chest and promoting lactation. After localized point massage of these acupoints, the milk can be smoothly discharged. Combined with localized breast massage, it promotes lactation and discharges accumulated milk. During the lactation promotion process, the patient feels no pain. After the first lactation promotion and the first day of oral administration of Chinese medicine, many patients’ clinical symptoms are significantly reduced.

In the treatment of lactational mastitis, we encourage continued breastfeeding, only temporarily suspend breastfeeding on the affected side when purulent and bloody milk is found while lactation promotion. Once the purulent and bloody milk disappears, breastfeeding can be resumed.^[14]^ After many years of clinical observation, no adverse reactions such as infant colic, bloating, diarrhea, nausea, vomiting, or fever have been found. Some people believe that breast milk bacteria may participate in the maturation of the infant immune system, regulating natural and acquired immune responses.^[14]^ Infants who are breastfed may be exposed to certain bacteria that have a beneficial effect on diarrhea and respiratory system diseases, and may reduce the risk of other diseases such as diabetes or obesity.^[20]^

Through the clinical treatment and observation of these 3 cases, we found that the traditional Chinese medicine Gualou Xiaoyong soup combined with painless lactation promotion techniques has good clinical efficacy in the treatment of lactational mastitis. It can significantly reduce and eliminate the clinical symptoms of patients in the short term, and rapidly restore inflammatory indicators such as total WBC, neutrophils, CRP, and procalcitonin to normal levels. The patchy low echo area of the breast under B-ultrasound also disappears quickly. Therefore, we believe that this method is a good way to treat lactational mastitis and is worthy of clinical reference and research. However, this study has some limitations: The sample size is relatively small, and it is only a medical record reporting study; this study lacks a large sample of prospective controlled studies. Next, we will continue to collect relevant cases and conduct prospective case randomized controlled clinical studies.

## 4. Conclusion

The combination of Gualou Xiaoyong soup and painless lactation promotion techniques has good clinical efficacy in the treatment of lactational mastitis. It has the advantages of preventing the formation of breast lumps, shortening treatment duration, not reducing breast milk, not affecting breastfeeding, and without toxic side effects or pain for the patient.

## Acknowledgments

This paper was completed under the careful guidance and strong support of Dr Jin. Dr Jin has had an important impact on me with his rigorous and realistic academic attitude, high professionalism, conscientious, diligent work style and innovative enterprising spirit. Her profound knowledge, broad vision and sharp thinking gave me deep inspiration. At the same time, during the design of this paper, I also learned a lot about Chinese medicine, and my clinical skills have been greatly improved.

## Author contributions

**Data curation:** Huijun Ye.

**Formal analysis:** Yi Yang.

**Methodology:** Jiali Liu.

**Supervision:** Huiling Zheng.

**Writing – original draft:** Lihua Jin, Ruilan Li.
